# UNICOR-v, a Pan-Coronavirus Subunit Vaccine, Demonstrates Immunogenicity and Efficacy Against MERS-CoV Infection

**DOI:** 10.3390/vaccines14040288

**Published:** 2026-03-24

**Authors:** Megan E. Cole, Siân Jossi, Carly Dillen, Rachel Fanaroff, Matthew Frieman, Olga Pleguezuelos

**Affiliations:** 1 ConserV Bioscience Limited, Heyford Park Innovation Centre, Bicester, Oxfordshire OX25 5HD, UK; 2 Center for Pathogen Research, Department of Microbiology and Immunology, University of Maryland School of Medicine, Baltimore, MD 21201, USA; 3 Department of Pathology, University of Maryland School of Medicine, Baltimore, MD 21201, USA; rachel.fanaroff@som.umaryland.edu

**Keywords:** pan-coronavirus, peptide, vaccine, MERS, T cells, antibodies, efficacy, preclinical

## Abstract

Background/Objectives: Coronaviruses are a family of positive-sense RNA viruses that cause respiratory and gastrointestinal disease in mammals and birds. Their zoonotic nature and high mutability make them a pandemic threat. UNICOR-v is a pre-pandemic, pan-coronavirus vaccine composed of an adjuvanted mix of twelve synthetic peptides originating from conserved regions within Nsp12 and M coronavirus proteins containing clusters of predicted T-cell epitopes. Here, we evaluate the immunogenicity of UNICOR-v and its efficacy against Middle East Respiratory Syndrome-related coronavirus (MERS). Methods: Animals were vaccinated with an adjuvanted equimolar mix of UNICOR-v. Humoral and cellular immunogenicity were assessed 28 days later through ELISA and FLUOROSpot. Vaccine efficacy was assessed in a DPP4 knock-in (HDPP4-KI) mouse model where mice were challenged post-vaccination with a lethal or non-lethal dose of MERS-CoV-MA. Results: Vaccination with UNICOR-v induced high IgG titers in both mice and rabbits and cellular secretion of pro-inflammatory cytokines. Vaccination with UNICOR-v, or passive serum transfer, significantly reduced viral lung titers 4 days post-infection compared to placebo. Vaccination induced lower immune cell infiltration in the alveolar space and increased repair of the cells lining the major airways in vaccinated mice, translating to increased survival rate compared to placebo. Conclusions: These data demonstrate the ability of conserved T-cell epitopes to protect against MERS-CoV infection, supporting further characterization of the breadth of protection of UNICOR-v against other coronaviruses that affect humans and livestock, following a One Health approach to control this highly zoonotic family of viruses.

## 1. Introduction

Coronaviruses are zoonotic viruses that can infect mammals and birds [1]. The *Orthocoronavirinae* subfamily [2,3] is composed of four genera, α-, β-, γ-, and δ-coronavirus [1,2], with the β-coronavirus genus including viruses that infect humans causing common cold (HCoV-OC43 and HCoV-HKU1), severe acute respiratory syndrome (SARS-CoV-1 and SARS-CoV-2), and Middle East Respiratory Syndrome (MERS-CoV) [4].

Viruses that infect vertebrate animals can sometimes spill over and infect humans (zoonosis). Lack of pre-existing immunity in humans to zoonotic viruses can lead to epidemics or pandemics [5], as experienced with the SARS-CoV-2 virus (COVID-19), which caused vast social [6], economic [7,8], and health impacts [9,10,11] worldwide. Many low- and middle-income countries (LMICs) are likely to be the epicenter of a new zoonotic epidemic due to humans living in close proximity to animals [12,13,14], having limited access to veterinary services and diagnostics, and lacking appropriate infrastructure around animal health and the meat trade. The wildlife trade in LMICs is also a lucrative unregulated business and a likely route for transmission of viruses from natural reservoirs, such as bats, to humans [15,16,17]. Coronaviruses also have a significant impact on animal health, causing severe gastrointestinal disease in young piglets and calves [18,19] and respiratory tract infections in poultry [20,21], which in turn carry their own economic burden, disproportionately affecting the livelihood of farmers in LMICs.

An effective and affordable vaccine that protects humans and animals could be of great benefit, especially in high-risk areas. A ‘One Health’ approach addresses the interconnectedness of human and animal health, a topic that has been brought to the forefront of disease prevention strategies as climate change and globalization increasingly affect the ecosystems shared by humans and animals [22]. The WHO has advocated for a One Health approach to tackle the threat posed by coronaviruses [23] given the wide range of hosts and high risk of zoonosis. A pan-coronavirus vaccine that could be used to protect humans and animals against existing and emerging strains is a prime example of such an approach. In the event of a novel strain emerging, existence of a broadly protective vaccine would reduce the need for implementation of severe measures, such as lockdowns, which have long-lasting societal implications [8,24]. In addition to efficacy, a pre-pandemic vaccine should also adhere to CEPI’s ‘100 day mission’ and comply with fast, flexible, and scalable manufacturing and global distribution [25,26,27].

Several approaches to achieving more broadly protective coronavirus vaccines are in development, including presentation of antigens from multiple strains within nanoparticles [28,29,30] or administration of antigens from different strains in serial vaccinations [31], although this approach could lead to vaccination fatigue. Others have opted to concentrate on the β-coronavirus genus only, as this genus contains the viruses that have caused epidemics or pandemics in humans so far [32,33,34]. More ambitious approaches are aiming for true pan-coronavirus vaccines [35] by generating broadly protective neutralizing antibody responses, although these vaccines have yet to demonstrate efficacy in humans. Here, we propose a different approach, UNICOR-v, a peptide-based vaccine that aims to provide protection against all viruses within the *Orthocoronavirinae* subfamily by targeting short, conserved regions of internal viral proteins. Using short peptides for vaccination instead of whole proteins allows the immune system to focus only on areas of high conservation that often include subdominant peptides that would otherwise be hidden to the immune system. This enables the generation of a broader response that may perhaps be less efficacious than that of a virus-specific vaccine but could be considered as the first line of attack in the event of a new coronavirus emerging. This approach would offer some protection in the population to avoid rapid spread of the virus and severe disease, thus avoiding lockdowns and hospitalizations, and could also provide valuable time to enable the development and distribution of a better, more efficacious and specific vaccine. In this manuscript, we present preclinical immunogenicity and proof-of-concept efficacy data using a highly pathogenic MERS-CoV mouse model as an example of an *Orthocoronavirinae* virus, thus providing the basis to further explore efficacy against other *Orthocoronavirinae* viruses in the future and characterize the potential breadth of protection of this vaccine.

## 2. Materials and Methods

### 2.1. Identification of Conserved T-Cell Reactive Sequences

CLUSTAL omega [36] was used to perform multiple sequence alignments for each viral protein in the *Orthocoronavirinae* proteome. A consensus sequence was generated from each alignment of all *Orthocoronavirinae* sequences available in the NBCI database. Regions within the consensus sequence with a minimum of 70% consecutive amino acid conservation were analyzed for the presence of reactive T-cell epitopes for multiple human HLA-I alleles and mouse MHC-I using a proprietary algorithm (PepTcell Ltd. (London, UK), parent company of ConserV Bioscience Ltd. (Bicester, UK)). The algorithm analyzes the structural affinity of a peptide for a particular HLA/MHC allele and uses data, extracted from a set of reactive vs. non-reactive epitopes identified empirically, to determine if a sequence of amino acids contains a ‘T-cell reactive’ or ‘self’ epitope for that HLA. The final selection of conserved polyepitope T-cell reactive peptide regions was made based on the length (min 20 amino acid (AA)-max 40AA), the presence of clusters of epitopes for all HLA alleles analyzed (at least one epitope/HLA/MHC), and not sharing similarity to other murine/human protein sequences in a BLAST (+ 2.12.0) search [37].

Conserved regions with a high density of predicted epitopes were selected and manufactured as acetate peptides (Genscript, Oxford, UK).

### 2.2. Animals and Procedures

Staff at the animal facilities were blind to the treatments throughout. Animals were randomized to a group while ensuring equal weight distribution of the animals in the group.

For immunogenicity studies, n = 4 was used for each group. This small group size is sufficient to generate reliable immunogenicity preliminary data while minimizing the number of animals used, in line with the 3R principles.

All murine work was performed at the University of Leicester (UK) Preclinical Research Facility managed by the Division of Biomedical Services. The purpose-built facility operates under the UK Home Office license to AMC (P6DCE1A76, 6 August 2018) under an establishment license to the University of Leicester (X1798C4D2). Seven- to nine-week-old female C57BL/6 mice bred in-house or C57BL/6 HLA0201 transgenic mice purchased from Charles River UK (license obtained from the University of Virginia, Charlottesville, VA, USA) were used in these studies.

Viroclinics Biosciences B.V. (Schaijk, The Netherlands) performed the immunogenicity studies in 10–14-week-old female New Zealand white rabbits purchased from Charles River, Germany. Overall approval for performing animal experiments related to intervention strategies against coronaviruses was given by the Central Authority for Scientific Procedures on Animals (Centrale Commissie Dierproeven) and registered under AVD27700202114492. The study was also approved by the internal Animal Welfare Body under registration number AVD27700202114492-WP41 on 8 May 2022. The animal studies were conducted under conditions that meet the standard of Dutch law for animal experimentation and agreed with the ‘Guide for the care and use of laboratory animals’, ILAR recommendations, and OLAW and AAALAC standards.

All animals were vaccinated subcutaneously twice, two weeks apart. Two weeks after the booster vaccination, animals were euthanized, and spleens and terminal blood were collected to measure immune responses to the vaccines.

MERS-CoV efficacy studies were conducted in the AAALAC-accredited animal facilities at the University of Maryland, Baltimore (USA), and approved by the Institutional Animal Care and Use Committee (IACUC) of the University of Maryland (Baltimore, MD, USA) (Code: AM-AUP-00000349-21, 17 November 2023). Breeding pairs of human DPP4 knock-in (hDPP4-KI) mice were kindly provided by Dr. Stanley Perlman from the University of Iowa (Iowa City, IA, USA). Mice had exons 10–12 of the MERS-CoV receptor, human DPP4, inserted into the mouse DPP4 receptor to increase susceptibility to MERS-CoV [38]. Mice were bred in house and vaccinated as described in the immunogenicity section (n = 10/group, 50:50 M:F 6–8 weeks). The sample size allowed for detection of a statistically meaningful effect on survival while reducing animal usage. Two weeks after the booster vaccination, mice were infected with mouse-adapted MERS-CoV-MA, kindly provided by Dr. Stanley Perlman from the University of Iowa (USA). The MERS-CoV virus initially replicated in hDPP4 knock-in mice but without disease. After serial passage in hDPP4 knock-in mice, the resultant mouse-adapted virus (MERS-CoV-MA) caused fatal lung disease that included diffuse alveolar damage and immune dysregulation. The virus was expanded in Vero E6-TMPRSS2 (VeroT) cells (ATCC, Manassas, VA, USA) and stocked at high-titer concentrations. The VeroE6/TMPRSS2 cell line was created from VeroE6 cells that were modified to constitutively express the human serine protease TMPRSS2 (Genbank# NM_005656.4) initially established with Geneticin (G418) (1 mg/mL) selection and now maintained in 10% FBS-DMEM, penicillin (100 unit/mL), and streptomycin (100 ug/mL). The resulting VeroE6/TMPRSS2 cells were shown to be highly susceptible to MERS-CoV infection and can be used to produce large viral stocks. The cell line originates from female *Chlorocebus aethiops*—Grivet monkey. The depositor is Dr. Makoto Takeda, who donated the cell line from the Japanese Collection of Research Bioresources Cell Bank (JCRB), National Institutes of Biomedical Innovation, Health and Nutrition (cell line #JCRB1819). The virus was sequenced-confirmed for fidelity. All mouse viral inoculations were carried out in a BSL-3 facility in accordance with approved practices. Mice were anesthetized with an intraperitoneal injection of a mixture of ketamine (Covetrus, Portland, ME, USA) and xylazine (Dectra, Overland Park, KS, USA) in sterile PBS (Corning Life Sciences, Tewksbury, MA, USA). While anesthetized, mice were intranasally inoculated with 50 uL of either sterile PBS or 5 × 10^3^ PFU of MERS-CoV-MA. Mice were monitored daily for weight loss and signs of morbidity. For the studies to evaluate viral titre, n = 5 mice in each group were euthanized 2 days post-inoculation and n = 5 mice were euthanized 4 days post-inoculation. Lungs were collected for further analysis of viral titer and histopathology. For the studies to measure survival (n = 10/group), mice were weighed daily until day 12 post-infection.

### 2.3. Immunizations

Peptides were manufactured by Genscript (Oxford, UK). Lyophilized peptides were reconstituted to a concentration of 20 mM in dimethyl sulfoxide (DMSO, Sigma-Aldrich, Gillingham, UK)) or water (Sigma-Aldrich, Gillingham, UK) as per the manufacturer’s recommendations. UNICOR-v was made up as an equimolar mix of the 12 peptides to reach concentrations of 5 nmol, 10 nmol, 20 nmol, or 30 nmol of each peptide in a 0.05 mL volume that was then mixed with 0.05 mL of adjuvant Montanide ISA-51 (water in oil emulsion adjuvant composed of a white mineral oil and mannide monooleate as non-ionic surfactant) (Seppic, Castres, France) immediately prior to dosing to create an emulsion.

Two vaccine controls were used, a 0.1 mL PBS subcutaneous injection and an adjuvanted placebo subcutaneous injection made up of 50 uL of water and 50 uL of Montanide ISA-51 emulsified. Mice were immunized twice two weeks apart as a subcutaneous injection between the shoulder blades. The same injection site was used for priming and boosting.

Rabbits were vaccinated following the same vaccination protocol as for mice but with two doses of 30 nmol of each peptide in a 0.3 mL volume.

### 2.4. Passive Serum Transfer

Blood was collected via cardiac puncture from vaccinated mice two weeks after receiving the booster dose. Blood was collected into a CAT-GEL serum isolation tube (Starstedt, Inc., Newton, NC, USA) and spun at 1000× *g* for 10 min, after which serum was removed into a sterile labelled tube. Sera from five mice was pooled and stored at +4 °C until use. Two hours prior to infection with MERS-CoV, mice in the serum transfer groups were injected intraperitoneally with 200 uL of serum using an ultra-fine insulin syringe (Becton, Dickinson and Company, Franklin Lakes, NJ, USA).

### 2.5. Measurement of Cellular Immune Responses

Splenocytes were isolated by pressing the spleens through a 70 um cell strainer (Thermo Fisher Scientific, Waltham, MA, USA). After centrifugation for 5 min at 200× *g*, the cell pellets were resuspended in 1 mL of red blood cell lysis buffer (Sigma-Aldrich, Gillingham, UK) and incubated for 1 min, followed by the addition of 1 mL of fetal calf serum (FCS, Sigma-Aldrich, Gillingham, UK). Splenocyte cell suspensions were washed three times with RMPI-1640 (Gibco, Loughborough, UK) + 1% penicillin/streptomycin (Sigma-Aldrich, Gillingham, UK) + 2 mM L-Glutamine (Sigma-Aldrich, Gillingham, UK) before counting viable cells using a hemocytometer.

#### 2.5.1. FLUOROSpot

Splenocytes (4 × 10^5^ cells/well) were added to 96-well FLUOROSpot plates (MabTech, Nacka Strand, Sweden) pre-coated with IFN-g, IL-2, and TNF-a capture antibodies, as per the manufacturer’s instructions, and incubated with a pool of the 12 peptides (2 mM of each peptide) in complete media (RPMI-1680 media +10% FCS, media alone (negative control), or 5 ug/mL Concanavalin A (Sigma-Aldrich, Gillingham, UK) in complete media. Anti-CD28 antibody (MabTech, Nacka Strand, Sweden) was added to provide the necessary co-stimulatory molecules to support T-cell activation. Plates were incubated for 40–46 h at 37 °C and 5% CO_2_ before proceeding to detection of the analytes using fluorescently labeled antibodies and following the manufacturer’s instructions. Plates were left to air dry, and spot-forming units (SFU) were counted using the IRIS2 reader (MabTech, Nacka Strand, Sweden). SFUs for each stimulation were normalized for each animal by subtracting the SFU counts in the media-only wells (negative control). The counts were adjusted to present the number of SFU/million cells. The median of the group was calculated for statistical comparison.

#### 2.5.2. ELISA

Splenocytes (4 × 10^5^ cells/well) were added to a V-bottom 96-well tissue culture plate and incubated with 4 mM of each of the twelve peptides in UNICOR-v individually. Cells were incubated for 48 h at 37 °C and 5% CO_2_ before proceeding to quantify IFN-γ, IL-2, and TNF-α cytokines in the cell supernatants using an ELISAMax kit (Biolegend, London, UK) as per the manufacturer’s instructions.

### 2.6. Antibody Titers by ELISA

ELISA 96-well microtiter plates were coated overnight at +4 °C with 100 uL of 2 mM of each peptide for the pooled UNICOR-v peptides or 4 uM of each of the twelve peptides separately. Plates were washed twice with PBS + 0.05% Tween-20 (Sigma-Aldrich, Gillingham, UK) (PBS-T) before blocking for 1 h with 200 uL of 1% BSA (Sigma-Aldrich, Gillingham, UK) in PBS (BSA/PBS). Plates were washed 4 times with PBS-T and serum samples diluted 1:200 in BSA/PBS were added. After two hours of incubation, plates were washed 6 times with PBS-T, followed by the addition of 100 uL of primary antibody (Table 1) in BSA/PBS and incubation for 1 h. If a detection antibody was required, the plates were washed 5 times, and 100 uL of detection antibody (Table 1) was added and incubated for 1 h. Plates were washed eight times before adding 100 uL of TMB solution (Biolegend, London, UK) and allowed to develop for 30 min before the addition of 100 uL of 1N H_2_SO_4_ (Fisher Scientific, Loughborough, UK). Absorbance in each well was read at 450 nm and corrected at 570 nm using the Thermo Multiskan EX (Thermo Electron Corporation/Fisher Scientific, Loughborough, UK). The absorbance in the blank wells (no sera) was subtracted from the corrected absorbance. The median absorbance was calculated per group for statistical comparison.

### 2.7. Histology

Lung sections were fixed in 4% paraformaldehyde (PFA, Thermo Fisher Scientific, Waltham, MA, USA) in phosphate-buffered saline (PBS, Corning Life Sciences, Tewksbury, MA, USA) for a minimum of 48 h, after which they were sent to the Histology Core at the University of Maryland, (Baltimore, MD, USA), for paraffin embedding and sectioning. Five-micrometer sections were prepared and used for hematoxylin and eosin (H&E) staining by the Histology Core Services. Sections were imaged at 10× magnification, and representative images were chosen. Figures were put together using Adobe Photoshop 2025 (version 26.0) and Illustrator software 2026 (version 30.0) (San Jose, CA, USA). H&E stained slides were viewed by a pathologist blinded to the treatments each group received. Light microscopy was used to view the slides, and inflammation was evaluated in comparison to an uninfected control lung section. Inflammation was separated into that involving the major airways (peribronchiolar), that involving the blood vessels (perivascular), and overall inflammation in the interstitial space. Each component was assessed semi-quantitatively on a 0–3 scale based on overall burden and extent of involvement (0 = no increased inflammation; 1 = mild overall inflammation and/or ≤2 number of inflammatory foci; 2 = moderate overall inflammation and/or 3–4 number of inflammatory foci; 3 = marked overall inflammation and/or ≥5 number of inflammatory foci).

### 2.8. Lung Viral Titration by Plaque Assay

Vero E6-TMPRSS2 (VeroT) cells (ATCC, Manassas, VA, USA) were cultured in DMEM (Sigma-Aldrich, St. Louis, MO, USA) supplemented with 10% (*v*/*v*) heat-inactivated fetal bovine serum (Sigma-Aldrich, St. Louis, MO, USA), 1% (*v*/*v*) penicillin/streptomycin (Sigma-Aldrich, St. Louis, MO, USA), and 1% (*v*/*v*) L-Glutamine (2 mM final concentration) (Sigma-Aldrich, St. Louis, MO, USA). Cells were maintained at 37 °C, 5% CO_2_.

MERS-CoV lung titers were quantified by homogenizing mouse lungs in 1 mL of phosphate-buffered saline (PBS) using 1.0 mm glass beads and a Bead-ruptor (Omni International, Kennesaw, GA, USA). Vero cells were plated in 12-well plates with 1.5 × 10^5^ cells per well. MERS-CoV virus titer in plaque forming units was determined through plaque assay. In the plaque assay, 25 uL of the lung homogenate was added to 225 uL of PBS and diluted 10-fold across a 6-point dilution curve with 200 uL of diluent (Corning Life Sciences, Tewksbury, MA, USA) added to each well. After 1 h, a 2 mL semi-solid agar (Thermo Fisher Scientific, Waltham, MA, USA) overlay containing DMEM was added to each well. Plates were incubated for 2 days at 37 °C (5% CO_2_) before plaques were counted manually by the investigator.

### 2.9. Statistical Analysis

All statistical analyses and representations were carried out using GraphPad Prism (Version 11.0.0) (San Diego, CA, USA). All statistical tests performed are documented in the figure legends. The cutoff value used to determine significance was *p* ≤ 0.05 for all tests. Normality testing was performed using the Shapiro–Wilk test.

## 3. Results

### 3.1. Antigen Selection

The stepwise analysis to select the vaccine antigens was carried out during 2020 and initially included all available viral sequences in NCBI from the a, b, g, and d genera of the *Orthocoronavirinae* subfamily of viruses. Due to the large number of sequences available for SARS-CoV-2 in the middle of the COVID-19 pandemic, duplicated sequences were removed before performing the sequence alignments to avoid bias and obtain a true representation of the entire subfamily. Our astringent analyses identified conserved immunogenic regions that met our selection criteria in only two viral proteins, the RNA-dependent RNA-polymerase (NSp12) and the membrane (M) protein. For NSp12, 340 sequences were aligned, and several regions of conservation were identified in the consensus sequence; however, only one contained predicted T-cell reactive epitopes. Because there was still some variation within the selected region, a decision was made to select three different amino acid sequences to ensure viruses from all four genera were represented (Table 2).

For the membrane protein, the analysis revealed that the level of amino acid sequence variation was still too high when sequences from the four genera, a, b, g, and d, were included in the alignments. Therefore, the analysis was repeated by separating the sequences from the four different genera. The alignments were initiated with a total of 9679 sequences, of which 5593 sequences corresponded to the SARS-CoV-related virus (now known as SARS-CoV-2). The sequences were separated as originating from a viruses (n = 1908), b (n = 6646), g (n = 793), and d coronaviruses (n = 190). For the b genera, the alignment was conducted with all b sequences and again with the b sequences excluding SARS-related sequences to remove bias in the alignments. As a result of these alignments, several areas of conservation were identified and inputted into our proprietary T-cell prediction algorithm. A total of nine peptides were selected for the membrane protein to ensure full coverage across the four genera (Table 2).

### 3.2. UNICOR-v Immunogenicity

**Figure 1:**
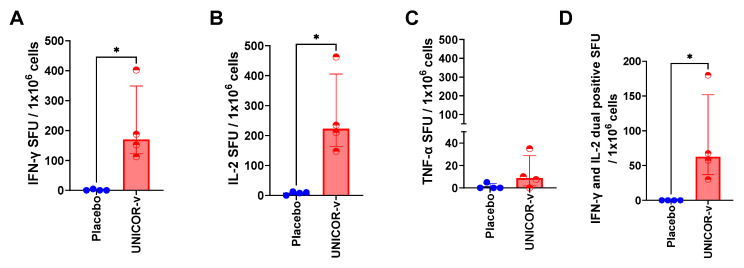
**Cytokine secretion by splenocytes after ex vivo exposure to UNICOR-v antigens**. C57Bl/6 mice received two doses of 10 nmol of UNICOR-v adjuvanted with Montanide ISA-51 (Seppic) (red) or adjuvanted placebo (blue), two weeks apart, as a subcutaneous injection (n = 4/group). Mice were culled two weeks post-boost and spleens were isolated. Splenocytes were exposed to the vaccine peptides, and secretion of (**A**) IFN-g (**B**), IL-2 (**C**), and (**D**) TNF-a was measured through FLUOROSpot assay. Double-positive cells secreting both IFN-g and IL-2 (**D**) were also quantified. Spot-forming units (SFU) were normalized for each animal to the SFU in unstimulated control wells. The graphs represent the SFU per 1 × 10^6^ splenocytes for each individual mouse (dots), and bars represent the group median and interquartile range. The Mann–Whitney test was used to compare the medians in the UNICOR-v vaccinated group and the placebo group, where * = *p* < 0.05.

Immunogenicity of 10 nmol adjuvanted UNICOR-v was assessed in C57BL/6 mice. This dose was chosen based on experiments with other peptide vaccines in ConserV Bioscience’s portfolio. Secretion of IFN-g, IL-2, and TNF-a cytokines by splenocytes exposed ex vivo to the pool of UNICOR-v antigens was measured through FLUOROSpot and used as a measurement of T-cell activation. The median number of IFN-g (Figure 1A) and IL-2 (Figure 1B) spots/million cells was significantly higher in vaccinated mice compared to the placebo (171-fold increase *p* = 0.0286 and 35-fold *p* = 0.0286 for IFN-γ and IL-2, respectively). Very little splenocyte secretion of TNF-a cytokine was detected in either of the groups (Figure 1C). Further analysis revealed that the median number of dual-positive cells secreting both IFN-g and IL-2 was 84-fold higher in the UNICOR-v vaccinated group than in the placebo (*p* = 0.0286) (Figure 1D), indicating polyfunctionality of the activated T cells.

**Figure 2:**
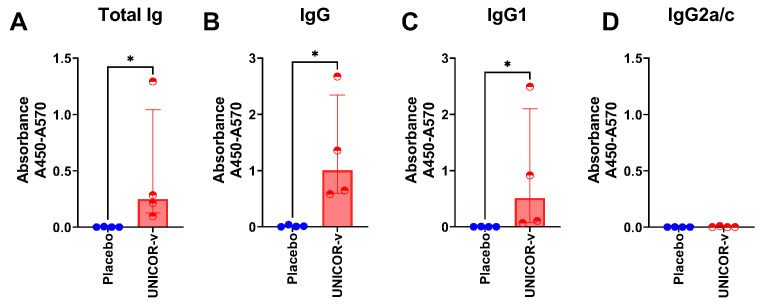
**Antibody responses to UNICOR-v antigens in mice.** C57Bl/6 mice received two doses of 10 nmol of UNICOR-v adjuvanted with Montanide ISA-51 (Seppic) (red) or adjuvanted placebo (blue) as a subcutaneous injection two weeks apart (n = 4/group). Mice were culled 2 weeks post-boost, and serum samples were collected to measure UNICOR-v-specific total immunoglobulin (Ig) (**A**), total IgG (**B**), IgG1 (**C**), and IgG2a/c (**D**) subclasses through ELISA. Absolute absorbance values were normalized to plate background (Abs450-Abs570 nm). Individual data points (dots) represent corrected absorbance for each mouse, and bars represent the group median with the interquartile range. The Mann–Whitney test was used to compare the median absorbances for each antibody class and subclass in the UNICOR-v vaccinated group and the placebo group (* = *p* < 0.05).

Selection of the vaccine antigens was conducted based on predicted T-cell reactivity. Nevertheless, because in other vaccine projects using the same platform peptides also induced antibody responses, we proceeded to measure UNICOR-v-specific antibodies in vaccinated mice. Vaccination induced moderate antibody responses in C57BL/6 mice that were significantly higher than in mice in the placebo group (Figure 2). Two doses of UNICOR-v were able to induce antibody class switching as significantly higher titres of IgG antibodies were detected in immunized mice compared to placebo mice (*p* = 0.0286) (Figure 2A,B). Furthermore, antibody subclass analysis revealed that the IgG response is more biased towards the IgG1 subclass than the IgG2a/c subclass (Figure 2C,D), with the caveat that the assay was not quantitative.

**Figure 3:**
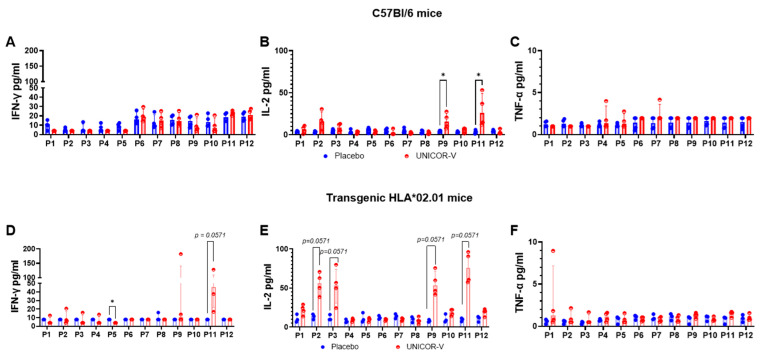
**Antigen-specific cellular responses induced after UNICOR-v vaccination.** C57Bl/6 mice (top row) or transgenic HLA02.01 mice (bottom row) received two doses of 10 nmol of UNICOR-v adjuvanted with Montanide ISA-51 (Seppic) (red) or adjuvanted placebo (blue) as a subcutaneous injection two weeks apart (n = 4/group). Animals were culled two weeks after the last vaccination, when spleens were collected. Splenocytes were exposed to the individual vaccine peptides, and ELISA was used to quantify the secretion of IFN-g (**A**,**D**), IL-2 (**B**,**E**), and TNF-a (**C**,**F**). The graphs represent the data for each individual mouse (dots), whereas bars represent the group median and the interquartile range. Multiple Mann–Whitney tests were used to evaluate differences between the median pg/mL secreted cytokines in the placebo and the UNICOR-v vaccinated groups (* = *p* < 0.05).

C57BL/6 mice transgenic for human leukocyte antigen 2.01 (HLA-A*2.01) were used to ascertain the type of immune response that could be expected in humans. In separate experiments, transgenic and non-transgenic C57BL/6 mice were immunized with adjuvanted UNICOR-v or adjuvanted placebo. Splenocytes were then exposed ex vivo to the individual peptides comprising UNICOR-v, and secretion of IFN-g, IL-2, and TNF-a cytokines in the cell supernatants was quantified through ELISA (Figure 3A–F). In this experiment, IL-2 was the cytokine secreted at the highest level by splenocytes from vaccinated mice, with the transgenic mice secreting higher levels (Figure 3E) than the wild-type mice (Figure 3B) in response to the UNICOR-v antigens. The higher activation observed in transgenic mice is thought to be the contribution of T-cell activation from antigens presented via human HLA-A*2.01 (Figure 3) in addition to presentation via murine MHC-I receptors. Stimulation of splenocytes with the individual peptides rather than the mix of the twelve peptides allowed us to determine that T-cell stimulation in mice is primarily due to the contribution of peptides P2, P3, P9, and P11.

**Figure 4:**
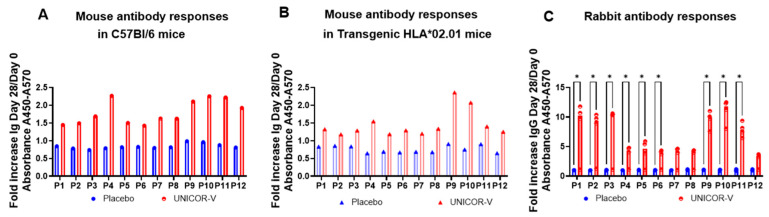
**Antigen-specific antibody titers induced after UNICOR-v vaccination.** C57Bl/6 mice (n = 4/group) or transgenic HLA02.01 mice (n = 4/group) received two doses of 10 nmol of UNICOR-v adjuvanted with Montanide ISA-51 (Seppic) (red) or adjuvanted placebo (blue) as a subcutaneous injection two weeks apart (n = 4/group). New Zealand white rabbits (n = 4/group) received 30 nmol of adjuvanted UNICOR-v following the same vaccination protocol as mice (n = 4/group). Serum samples were collected before vaccination (day 0) and two weeks after the last vaccination (day 28). Peptide-specific total immunoglobulin (Ig) was measured in C57Bl6 transgenic mice (**A**) and in C57Bl6 wild-type (**B**) using group-pooled sera. In rabbits, IgG titers were measured for each rabbit (**C**). Absolute absorbance values were corrected to plate background (450–570 nm), and the fold increase in absorbance from day 0 to day 28 was calculated (corrected absorbance day 28/corrected absorbance day 0). Graphs A-B represent the fold increase in titers in pooled sera in mice, whereas in (**C**) the dots represent the fold increase for each rabbit (dot) and the bars represent the median fold increase for the groups with interquartile ranges. Multiple Mann–Whitney tests were used to evaluate differences between the medians in the placebo and the vaccinated groups in rabbits (* = *p* < 0.05). No statistical analysis could be performed for mice, as a single measurement was obtained for each group.

Similarly, analysis of the antibody responses in the serum of mice demonstrated a similar response in transgenic and non-transgenic mice, with P9, P10, and P11 peptides inducing the highest antibody titers (Figure 4A,B). It is important to remark that C57BL/6 mice are more biased towards inducing cellular responses rather than antibody responses; hence, an additional study was conducted to determine the potential of UNICOR-v in inducing antibody responses in New Zealand white rabbits, a well-known animal model for exploring antigen-specific antibody responses. The data showed that all peptides induced robust antibody responses in rabbits, but these were also particularly stronger against peptides P1-P3 and P9-P11 (Figure 4C), mirroring the murine data but to a much higher level of response (5–10 fold increase in rabbits compared to 1.5–2 fold increase in mice).

### 3.3. UNICOR-v Efficacy Against MERS-CoV

Efficacy of UNICOR-v against MERS-CoV was evaluated in hDPP4-KI mice that were vaccinated with different doses of UNICOR-v (5, 10, 20, and 30 nmol), then challenged with a non-lethal dose of MERS-CoV-MA strain 2 weeks post-boost. Additionally, pooled serum from five mice vaccinated with UNICOR-v was injected intraperitoneally to naïve mice two hours prior to challenge to assess whether antigen-specific antibodies may contribute to protection. Two time points (2 days post-infection (dpi) or 4 dpi) were selected to evaluate differences in viral titers and lung pathology between the vaccinated groups and the placebo. Body weight was also monitored daily post-infection as a measurement of infection-associated morbidity.

#### 3.3.1. Weight

**Figure 5:**
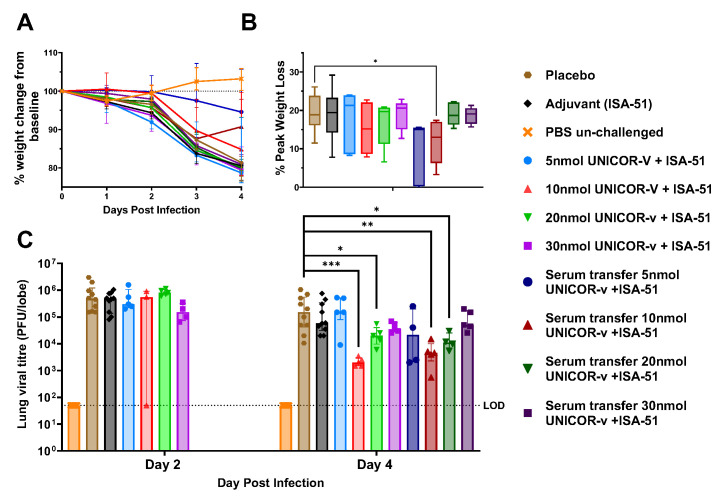
**Efficacy of UNICOR-v against non-lethal challenge with MERS-CoV-MA in hDPP4-KI mice.** Mice received two doses of 5, 10, 20, or 30 nmol of UNICOR-v adjuvanted with Montanide ISA-51 (Seppic), adjuvanted placebo, or PBS as a subcutaneous injection two weeks apart (n = 10/group). Two weeks post-boost mice were infected with 5 × 10^3^ PFU MERS-CoV-MA. In some groups, intraperitoneal vaccinated serum was given to naïve mice two hours prior to infection with MERS-CoV-MA (n = 5/group). Five mice per group in the vaccinated or placebo groups were euthanized on day 2 post-infection and the other five on 4 d.p.i. The mice in the serum transfer groups were all euthanized after 4 d.p.i. (**A**) Animals were weighed daily post-infection, and data are presented as the percentage weight change from baseline. Represented are the group median and interquartile range. (**B**) Peak weight loss compared to baseline. Boxes represent the median with 5–95% percentile. (**C**) Lung viral titers were quantified at days 2 and 4 post-infection. Significance of differences between the medians in vaccinated groups and placebo was evaluated using the Mann–Whitney, where * = *p* < 0.05, ** = *p* < 0.005, *** = *p* < 0.0005.

All challenged animals lost a similar amount of weight over the time course of the infection, with no significant differences between groups (Figure 5A). Analysis of peak weight loss revealed that mice receiving a serum transfer from mice vaccinated with 10 nmol UNICOR-v lost significantly less weight than animals in the placebo group (*p* = 0.0079) (Figure 5B). There were no differences in weight loss between any other group and the placebo.

#### 3.3.2. Lung Viral Titers

Animals in all MERS-CoV infected groups showed similar viral titres in the lungs at 2 dpi (Figure 5C). This is expected, as, for a T-cell-driven vaccine to act, cellular viral replication is required for T cells to detect an infected cell. In contrast, vaccines that rely on the production of neutralizing antibodies can offer early protection by neutralizing free extracellular virus, thus impeding cellular infection and proliferation. Therefore, the effects of a T-cell response are expected to be detected later than those from a neutralizing antibody response. Following from this, by 4 dpi mice vaccinated with 10 nmol or 20 nmol UNICOR-v displayed significantly reduced viral titers compared to the placebo group (*p* = 0.0007 and 0.044, respectively) (Figure 5C). This timepoint would be consistent with the induction of T cell responses. In addition, mice receiving passive transfer of serum from mice vaccinated with 10 nmol or 20 nmol of UNICOR-v also showed a significant reduction in viral titers (*p* = 0.0013 and 0.012, respectively) at 4 dpi. Because the antigens in UNICOR-v are located in regions of the viral proteins that are present inside of the virus, it is unlikely that any antibody generated by UNICOR-v vaccination will have neutralizing potential, and we hypothesize that the observed protective effect is possibly due to killing of infected cells through UNICOR-v-specific antibodies binding to cells and activating complement or antibody-dependent cellular responses.

#### 3.3.3. Histopathology

**Figure 6:**
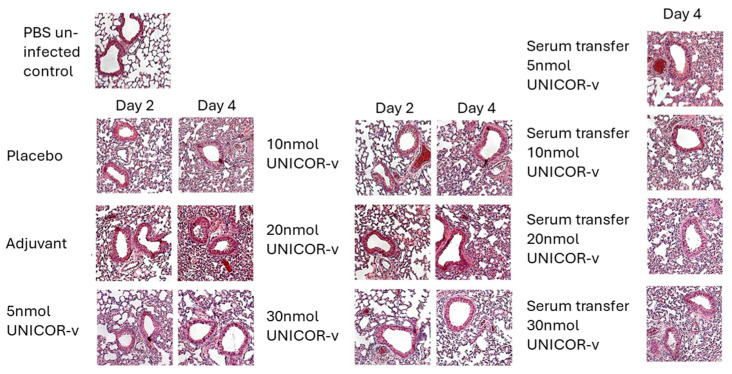
**Lung histopathology at days 2 and 4 post-MERS-CoV infection.** hDPP4-KI mice received two doses of 5, 10, 20, or 30 nmol of UNICOR-v adjuvanted with Montanide ISA-51 (Seppic), adjuvanted placebo, or PBS as a subcutaneous injection two weeks apart (n = 10/group). Two weeks post-boost, mice were infected with 5 × 10^3^ PFU MERS-CoV-MA. Mice in serum transfer groups received an intraperitoneal injection of UNICOR-v vaccinated sera two hours prior to infection with MERS-CoV-MA. Mice were euthanized at either day 2 or 4 post-infection (n = 5 per timepoint for vaccinated groups, n = 10 for sham/adjuvant groups). Lungs were isolated, and sections were H&E stained. Sections were imaged at 10× magnification. Representative H&E images shown.

H&E staining of lung tissue was performed to assess histopathology post-infection (Figure 6). Compared to the unchallenged PBS control mice, the lungs of challenged PBS placebo mice showed increased perivascular and interstitial inflammation. This was composed primarily of mononuclear cells. While occasional airways and alveolar spaces contained proteinaceous secretions or edema, respectively, peribronchiolar inflammation was sparse. These changes typically started at 2 dpi and progressed further by day 4 dpi. The same level of damage was observed in challenged mice given adjuvant alone.

Vaccination with any of the doses of UNICOR-v evaluated resulted in some improvement in inflammatory damage at both 2 and 4 dpi, particularly in the lungs of mice that received the 10 nmol or 30 nmol UNICOR-v doses. The lungs of these animals showed reduced cellular infiltration and wider, more intact airways. Interestingly, the 20 nmol dose had the most moderate effect on lung pathology despite significantly reducing viral titer (Figure 6).

**Figure 7:**
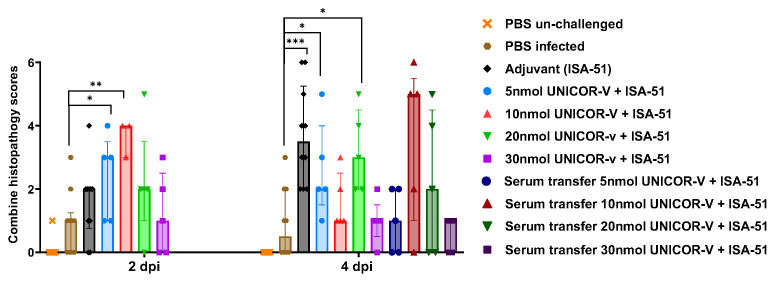
**Combined lung pathology scoring.** hDPP4-KI mice received two doses of 5, 10, 20, or 30 nmol of UNICOR-v adjuvanted with Montanide ISA-51 (Seppic), or adjuvanted placebo, or PBS as a subcutaneous injection two weeks apart (n = 10/group). Intraperitoneal serum transfer was performed 2 h prior to infection with MERS-CoV-MA. Two weeks post-boost mice were infected with 5 × 10^3^ PFU MERS-CoV-MA and culled at either day 2 or 4 post-infection (n = 5 per timepoint for vaccinated groups, n = 10 for sham/adjuvant groups). Lungs were fixed with 4% PFA and paraffin embedded before sections were H&E stained. Sections were imaged at 10x magnification and evaluated for severity of inflammation. Scores were then combined for each mouse and graphed. Differences between the medians in the vaccinated groups compared to the placebo were evaluated through Mann–Whitney test, where * = *p* < 0.05, ** = *p* < 0.005, *** = *p* < 0.0005.

When evaluating the combined histopathology scores at 2 dpi, the mice that received the 10 nmol dose had the highest score, indicating a high amount of infiltration. These marked early inflammatory changes may reflect rapid immune recall rather than immunopathology because the scores in these animals were drastically reduced by 4 dpi compared to the placebo (Figure 7). This suggests that the increased inflammation and infiltration observed on day 2 resulted in increased viral clearance by 4 dpi and consequent reduction in inflammation. In addition, all serum transfer groups showed similar histopathology to non-vaccinated mice, despite having induced reductions in viral titers in some groups.

#### 3.3.4. Survival in Lethal Study

**Figure 8:**
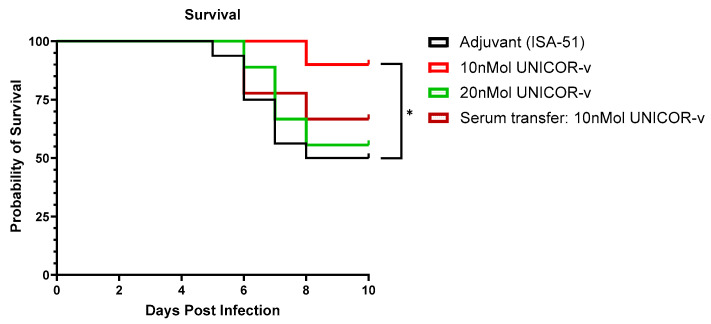
**Efficacy of UNICOR-v after challenge with LD50 MERS-CoV-MA.** hDPP4-KI mice received two doses of 10 or 20 nmol of UNICOR-v adjuvanted with Montanide ISA-51 (Seppic), or adjuvanted placebo, or PBS as a subcutaneous injection two weeks apart (n = 10/group). Intraperitoneal serum transfer was performed 2 h prior to infection with MERS-CoV-MA. Two weeks post-boost mice were infected with 5 × 10^3^ PFU MERS-CoV-MA and followed for up to 10 days. Differences in the survival outcomes of animals in the treatment groups compared to placebo on day 10 post-infection were calculated (* *p* = 0.013 based on Mantel–Cox test).

To evaluate the efficacy of UNICOR-v vaccination in terms of survival, hDPP4-KI mice were vaccinated with either 10 nmol or 20 nmol of UNICOR-v, as these doses demonstrated the highest protective effect. Mice were then infected with the LD50 of MERS-CoV-MA and monitored for up to 10 dpi. In mice receiving adjuvanted placebo, dramatic weight loss was observed between days 2 and 4, and mice reached clinical end point from day 5 onwards due to rapid body weight loss (≥20%) and/or severe symptomatology (inability to eat or drink, severe lethargy or prostration, and labored breathing). A similar pattern of weight loss was seen across vaccinated groups, confirming all mice were successfully infected, with no significant differences in peak weight loss between any group (Supplementary Figure S1A,B). However, vaccination with 10 nmol of UNICOR-v showed significant improvement in survival (*p* = 0.013), with 90% of animals still surviving on day 10 post-infection (Figure 8). Interestingly, vaccination with 20 nmol of UNICOR-v did not confer any improvement in survival compared with the adjuvant-only groups (Figure 8). Although a moderate effect on survival was seen in mice receiving a serum transfer from 10 nmol vaccinated mice, this was non-significant and highlights the importance of T cell responses to protect against infection (Figure 8).

## 4. Discussion

In silico predictions and analyses were used to identify and select the antigens composing UNICOR-v to generate a pan-*Orthocoronavirinae* vaccine. UNICOR-v is composed of twelve peptides originating from protein regions conserved across sequences in the *Orthocoronavirinae* subfamily of viruses. UNICOR-v was designed to induce T cell activation upon vaccination, with the aim of detecting infected cells by targeting internal conserved protein regions within the virus, thus offering broader protection. Our decision to use short peptides rather than whole proteins or the whole virus was motivated by the desire to remove proteome regions of low conservation, non-immunogenic regions, and regions that were similar to other human/murine protein sequences to avoid autoimmune responses. Following this approach, we expected to increase the signal to noise ratio, focusing the immune response on regions more likely to induce long-lasting, broader responses. In our studies, vaccination with UNICOR-v induced cellular responses in mice, characterized by activation of cells capable of secreting pro-inflammatory cytokines, in particular IFN-g and IL-2. Both cytokines are recognized as markers of T cell activation, with IL-2 inducing proliferation and differentiation of T cells and IFN-g inducing Th1 polarization necessary to kill intracellular pathogens. Both C57BL/6 wild-type mice and HLA*02.01 transgenic mice followed a similar pattern of immunological responses to UNICOR-v vaccination, although the response in the transgenic mice was of a higher magnitude, likely due to additional antigen presentation through the human HLA receptor in addition to the murine MHC receptor. This increase in the response suggests that UNICOR-v could also trigger an immune response in humans.

Despite the vaccine design used not aiming for induction of antibody responses, other vaccines developed using the same platform have previously demonstrated induction of non-neutralizing antibody responses capable of activating NK cells [39]. Therefore, we were also interested in evaluating whether UNICOR-v was able to trigger humoral responses. Vaccination with UNICOR-v induced antibody responses with major contributions from the IgG1 subclass. This discovery led to a modification in the planned efficacy studies to introduce additional groups to evaluate the protective effect of administration of serum from UNICOR-v vaccinated mice prior to viral challenge.

Further characterization of immune responses to the individual peptides revealed differences in the strength of response induced by each individual peptide in mice. Notably, peptides 4–8 gave poor overall responses in mice. To address this, the individual peptide responses were also evaluated in rabbits, a species that can mount an antibody response closer to that of humans due to their larger VDJ gene repertoire [40]. Vaccination of rabbits induced peptide-specific antibodies across all 12 peptides, although peptides 4–8 continued to induce more modest responses compared to the other peptides in the mix, as was also observed in mice. These data warranted the inclusion of all twelve peptides in the final UNICOR-v mix, as a similar response to rabbits could also be expected in humans.

The difference in the magnitude of the immune response between the two species used, mice and rabbits, highlights the limitations of using murine models; while useful for initial assessment of immunogenicity and efficacy, future studies should be expanded to include more relevant species that could be carriers and transmitters of coronaviruses, such as poultry, pigs, and camels. This would not only enable preclinical evaluation of vaccine safety, immunogenicity, and efficacy prior to evaluation in humans but would also contribute to a One Health approach. A vaccine that protects not only humans but the virus reservoir too would generate a barrier between them, reducing infections in humans. This was perfectly exemplified for rabies, where vaccination of canines in several countries reduced the number of human rabies cases dramatically [41,42].

To determine whether the immune responses to UNICOR-v observed in mice had the potential to be protective, vaccinated mice were challenged with MERS-CoV-MA. Mice receiving 10 nmol of adjuvanted UNICOR-v experienced lower viral titres on day 4 pi, which led to increased survival compared to the placebo. This is a remarkable achievement given the highly pathogenic nature of the strain of virus used in this challenge model. No differences in viral titers were observed in vaccinated mice 2 dpi compared to placebo mice. This delay in inducing protection is consistent with protection induced by activation of T cells, which relies on recognition of virus-infected cells compared to neutralizing antibodies that can recognize a free virus in circulation and therefore induce faster protection. T cell responses and non-neutralizing antibodies aid with viral clearance by destroying infected cells, reducing the spread of the infection and thus lessening the severity of disease [43,44]. Upon antigen recognition, T cells can release cytotoxic granules containing perforin and granzymes, which can trigger apoptosis [45], and produce cytotoxic cytokines, such as TNF-α and IFN-γ [46]. The magnitude and the diversity of the cellular immune response are key factors in determining how productive the immune response will be to the antigen in question [47]. Activation of T cells that secrete multiple cytokines has been associated with more effective cellular responses that are able to induce cell proliferation, modulate inflammation, mediate cytolysis of other cells, and inhibit viral replication [48]. Analysis of the cellular responses to UNICOR-v identified a population of cells that secreted both IL-2 and IFN-γ. It would be of interest to use techniques such as single cell analysis to understand the relationship between the timing of the secretion of each cytokine and the immune status of the cell [49]. In addition, to confirm that protection is driven by T cell responses and to what extent CD4+ T cells and CD8+ T cells are involved, challenge studies could be carried out where animals are depleted of a particular T cell type by means of using anti-CD4 or anti-CD8 antibodies.

Similar to the effect seen on viral titers in vaccinated mice, histopathology differences were also only observed on day 4 pi. It is, however, difficult to determine whether the pathology observed is caused by the virus or by the immune response to the virus. Further time course studies should be carried out to truly understand the succession of events and correlate the viral titers and cellular phenotype with the observed histopathology. Phenotyping of the infiltrating cells in the lungs will determine when immune cells reach the lungs and whether they are naïve T cells activated by the virus or vaccine-induced memory cells that provide a much faster response to the virus [50].

While epitopes were selected with the intent to induce T-cell responses, a moderate vaccine-specific antibody response was also detected. The antibody response is unlikely to be neutralizing as the peptides originate from internal proteins, but non-neutralizing antibodies bound to viral antigens on the surface of infected cells can contribute to viral clearance through opsonization of cells that leads to the activation of NK cells [51,52,53] and activation of complement via binding of C1q to the antigen–antibody complexes resulting in phagocytosis of the infected cell [54]. To determine if the antibodies induced by UNICOR-v vaccination contributed to vaccine efficacy, serum transfer from mice immunized with 10 or 20 nmol of UNICOR-v was injected in mice prior to the challenge. These animals had reduced viral lung titres post-infection; however, no improvement in lung histopathology or overall survival after lethal challenge was observed. This suggests that although the antibodies induced by UNICOR-v may reduce viral titer, cellular responses are necessary to achieve the significant increase in survival observed in vaccinated mice. Further work will be required to determine the mode of action of the UNICOR-v-specific antibodies in contributing to the observed effects in these studies.

One key finding from our studies was the different effects of the different doses of UNICOR-v. The 10 nmol dose was used in the immunogenicity mice studies based on the dose used in previous peptide-based vaccines studies [55,56]. The dose was increased to 30 nmol in rabbits to account for their larger body mass. Rabbits mounted a much stronger antibody response, so to ensure that this was a species-specific effect and not due entirely to dosing, a range of doses was evaluated in the efficacy studies in mice. A dose of 10 nmol consistently offered the greatest efficacy, but protection was variable at higher doses, with 30 nmol reducing lung histopathology but not viral titres and 20 nmol failing to improve survival. One potential explanation for this response is that higher doses of peptide may induce T cell exhaustion [57]. Repeated antigen exposure or sustained antigen persistence as a result of high dosing can overwhelm T cell responses and drive them to exhaustion, a differentiation state that actively inhibits cellular function [58,59]. In addition, lower antigen doses can potentially generate higher avidity T cells, which can detect smaller amounts of antigen present on cells in the earliest stages of infection [60,61]. This could explain why the 10 nmol dose induced higher viral clearance and better survival than the 20 or 30 nmol doses, and it is possible that the 5 nmol dose was below the activation threshold. Further analysis would be necessary to confirm this hypothesis by profiling the T cells with flow cytometry using markers of T cell exhaustion such as PD-1, CTLA-4, and TIM-3 accompanied by reduced IFNg, TNFa, and IL-2 production. In addition to this, examination of the vaccine formulation itself could elucidate different physical properties depending on the dose, which could affect how peptides interact with the adjuvant, resulting in different sizes and compositions of particles with different uptake patterns by antigen-presenting cells.

We set out to develop a pan-coronavirus vaccine and demonstrated its efficacy against MERS-CoV, a coronavirus member of the *Orthocoronavirinae* subfamily with a high mortality rate [62] and no current available licensed vaccine [63]. While several spike-based MERS-CoV vaccines are in development, three of which are in phase I clinical trials (NCT04170829, NCT04119440, and NCT03721718), none of them take into consideration that the spike protein is subject to immune pressures leading to antigenic drift across the different serotypes of MERS-CoV [63]. This can compromise the efficacy of a vaccine that relies on detection of the spike protein to provide cross-protection against new variants, as demonstrated with the SARS-CoV-2 COVID-19 vaccinations [64,65,66]. Unlike COVID-19 vaccines, the peptide sequences in UNICOR-v originate from viral proteins that have not been subjected to immune selection pressures over time and are therefore very conserved. An optimized UNICOR-v could potentially protect against emergent new strains or strains currently infecting other animals and spilling over into humans in the future.

We have previously demonstrated in clinical trials that the use of conserved peptide-based vaccines can successfully protect humans against viral infections such as influenza by inducing cross-reactive responses [67]. Future work with UNICOR-v will need to focus on exploring different methods to deliver the peptides to increase the magnitude of immune responses and to evaluate immunogenicity and efficacy in different species against other members of the *Orthocoronavirinae* subfamily. These data will elucidate the breadth of protection induced by UNICOR-v and its potential to prevent zoonotic epidemics.

## 5. Conclusions

Here, we demonstrate that UNICOR-v induces a multifaceted immune response that provides protection against MERS-CoV-MA infection, with 90% of mice surviving the challenge. The data warrant further research to improve the immune responses generated by UNICOR-v and to further investigate its efficacy protecting against other viruses within the *Orthocoronavirinae* subfamily. Further to this, evaluation of UNICOR-v’s efficacy in other species, such as camels, pigs, bovine, and poultry, will be an important step to address whether UNICOR-v could be an efficacious One Health vaccine.

## 6. Patents

Results from this work were used to submit a patent application: Coronavirus Immunogenic Compositions, Methods and Uses Thereof (US20220226465A1, WO2022152939A1, CA3208643A1, EP4277654A1, AU2022208435A1).

## Figures and Tables

**Table 1:** Immunoglobin isotyping antibodies.

Primary Antibody	Detection Antibody	Dilution
Goat anti-mouse IgG-HRP (BioLegend, London, UK)	N/A	1:2000
Goat anti-mouse Ig-HRP (Sigma-Aldrich, Gillingham, UK)	N/A	1:5000
Rat anti-mouse IgG1 (BioLegend, London, UK)	Goat anti-rat Ig-HRP (BioLegend)	1:1000 and 1:3000
Rat anti-mouse IgG2a/c (BioLegend, London, UK)	Goat anti-rat Ig-HRP (BioLegend)	1:500 and 1:3000

**Table 2:** UNICOR-v peptide sequences and protein of origin.

ID	Sequence	Protein of Origin
P1	TKRNVLPTMTQLNLKYAISGKERARTV	NSp12
P2	TKRNVIPTITQMNLKYAISAKNRARTV	NSp12
P3	TKRNVLPTLTQMNLKYAISAKNRARTV	NSp12
P4	PTGVTLTLLSGNLYAEGFK	Membrane
P5	PTGVTLTLLSGTLLVEGYK	Membrane
P6	PTGITVTLLSGVLYVDGHR	Membrane
P7	PTGVTLTLLSGVLLVDGHK	Membrane
P8	PTGITLTILSGTLFFDGIRIA	Membrane
P9	YFIASFRLFARTRSMWSFNPETN	Membrane
P10	YFVNSIRLFIRTGSWWSFNPETN	Membrane
P11	GYWIQSIRLFKRCRSWWSFNPESN	Membrane
P12	KLILLWLLQPFTLVVTIW	Membrane

## Data Availability

Archives of the data collected are not publicly available.

## Supplementary Materials

The following supporting information can be downloaded at https://www.mdpi.com/article/10.3390/vaccines14040288/s1, Figure S1: Efficacy of UNICOR-v after challenge with LOD_50_ MERS-CoV-MA.
