# Supplementary figures and images for: UNICOR-v, a Pan-Coronavirus Subunit Vaccine, Demonstrates Immunogenicity and Efficacy Against MERS-CoV Infection
Source: Vaccines (Basel). 2026 Mar 24;14(4):288. doi: 10.3390/vaccines14040288 (PMC13119802; doi:10.3390/vaccines14040288)

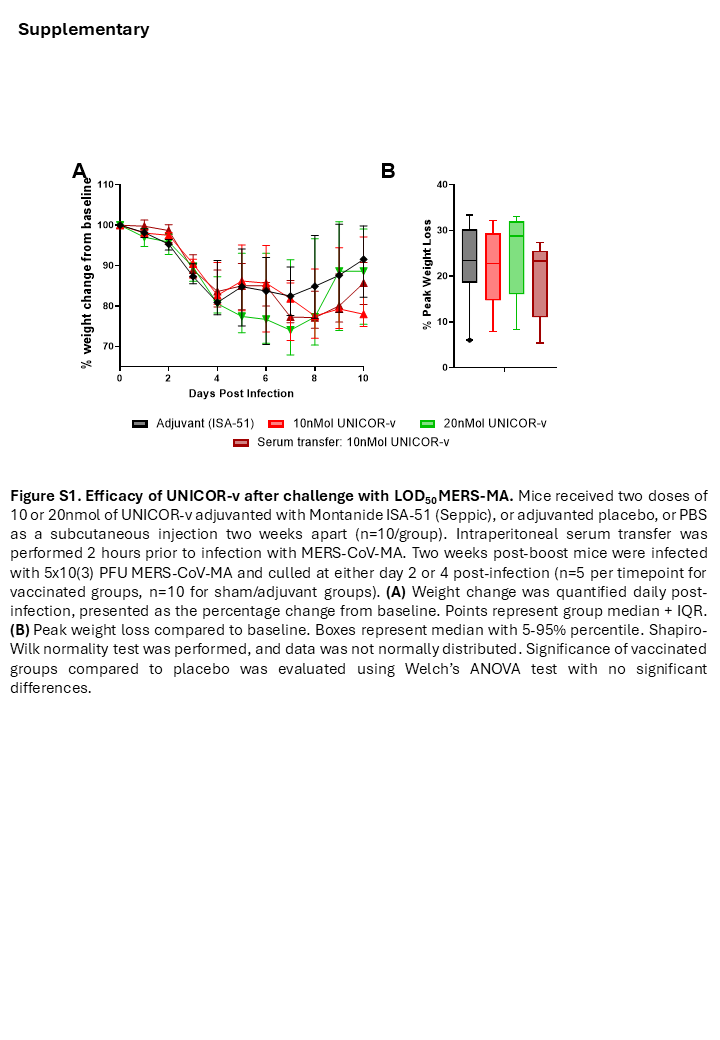

Supplement: Supplementary file 1 [file vaccines-14-00288-s001.zip › vaccines-4164686-supplementary.TIF]
